# Supplementary material for: Efficient mRNA Delivery In Vitro and In Vivo Using a Polycharged Biodegradable Nanomaterial
Source: Int J Mol Sci. 2024 Dec 19;25(24):13620. doi: 10.3390/ijms252413620 (PMC11728123; doi:10.3390/ijms252413620)
Supplement: Supplementary file 1 [file ijms-25-13620-s001.zip › ijms-3353384-supplementary.pdf]

Supporting Information

Efficient mRNA Delivery *In Vitro* and *In Vivo* Using a Polycharged Biodegradable Nanomaterial

Xuejin Yang [1,†], Jingya Xiao [1,†], Daryl Staveness [1], Xiaoyu Zang [1,*]

[1] N1 Life, Inc. 446 S Hillview Dr, Milpitas, CA 95035, USA

*Correspondence: X.Z., Janice@n1life.co

† These authors contributed equally to this work.

Table of Contents

Figure S1. Phase contrast and GFP fluorescent microscopy images of 18 cell lines transfected with N1-501/eGFP mRNA or Lipofectamine 3000/eGFP mRNA nanocomplexes. Pictures were captured 24 hours post-transfection (Scale bar, 400 µm)..........S3

Figure S2. Mean fluorescence intensities (MFI) of 18 cell lines treated with N1-501/eGFP mRNA and Lipofectamine 3000/eGFP mRNA complexes at a mRNA dose of 60 ng and N1-501 dose of 0.30 µL per well. The fluorescence images were captured at 4, 12, and 24 hours post-transfection. The MFI was calculated by dividing the total fluorescence by the number of GFP-positive cells..........S6

Figure S3. The correlation between cell viability and transfection efficiency was analyzed across 18 cell lines transfected with (A) N1-501/eGFP mRNA and (B) Lipofectamine 3000/eGFP mRNA nanoparticles for 24 hours. The summary statistics are shown in the tables below..........S7

Figure S4. Cell viability of HEK 293T treated with N1-501/eGFP mRNA nanoparticles formulated in different buffers, pH range, and media. Viability was evaluated using CCK-8 assay after 24 hours of treatment. S8

Figure S5. The shelf life of N1-501 was evaluated by transfection efficacy *in vitro*. HEK 293T cell viability was evaluated 24 hours after treatment with N1-501/eGFP mRNA nanoparticles using CCK-8 assay. S9

Table S1. Cell culture and seeding densities for the 18 cell lines evaluated in this study..........S10

Table S2. Cell viability of 18 cells treated with N1-501/eGFP mRNA, and Lipofectamine 3000/eGFP mRNA complexes for 24 hours, compared to negative control groups. All studies were conducted using an mRNA dose of 60 ng per well and evaluated with a CCK-8 assay..........S11

Table S3. Comparison of the size, polydispersity, and zeta potential of N1-501/eGFP mRNA nanoparticles prepared via pipette mixing and shaker mixing methods. The measurements were conducted using the Malvern Zetasizer Pro..........S12

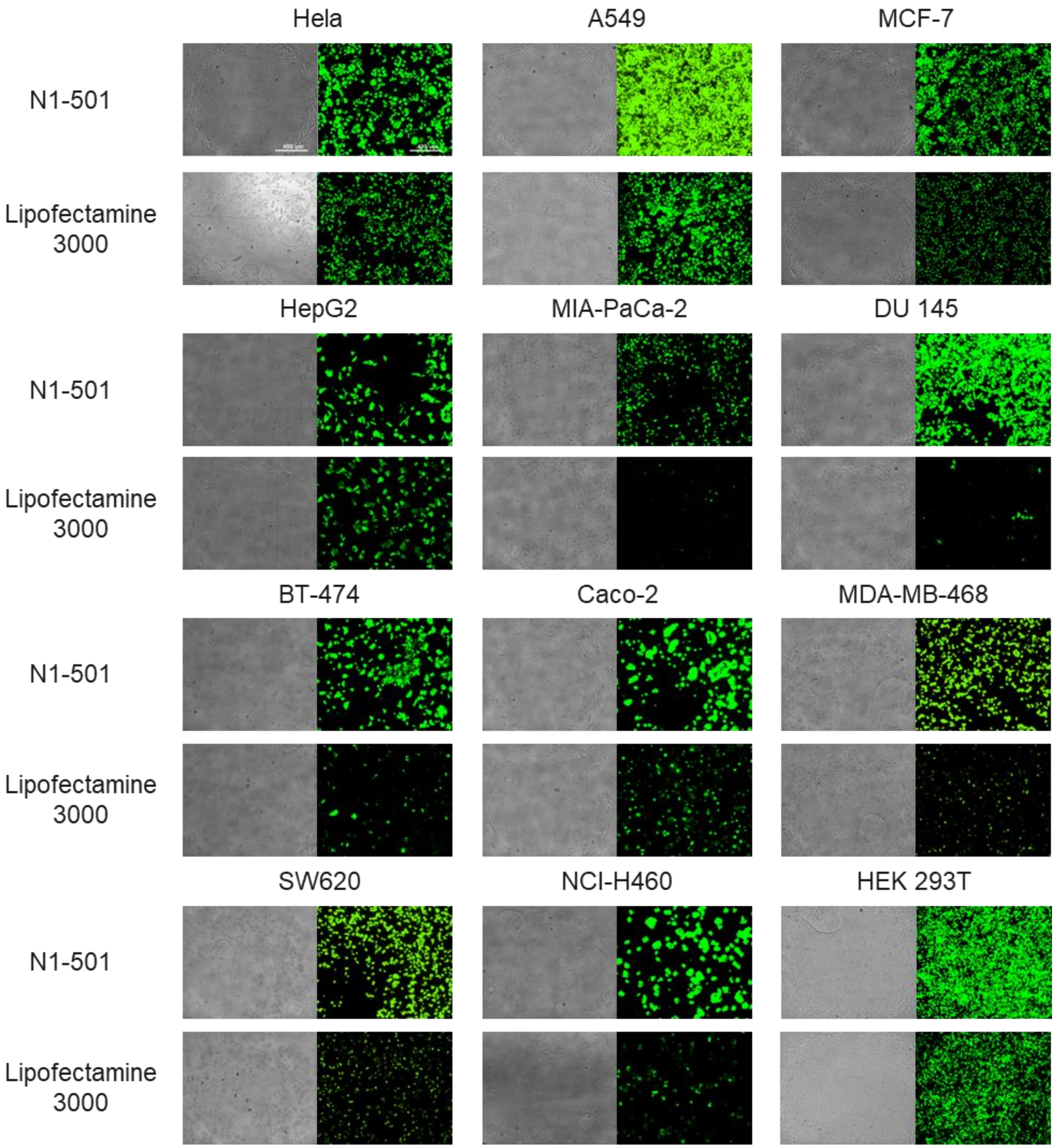
Hela
A549
MCF-7
N1-501
Lipofectamine 3000
HepG2
MIA-PaCa-2
DU 145
N1-501
Lipofectamine 3000
BT-474
Caco-2
MDA-MB-468
N1-501
Lipofectamine 3000
SW620
NCI-H460
HEK 293T
N1-501
Lipofectamine 3000

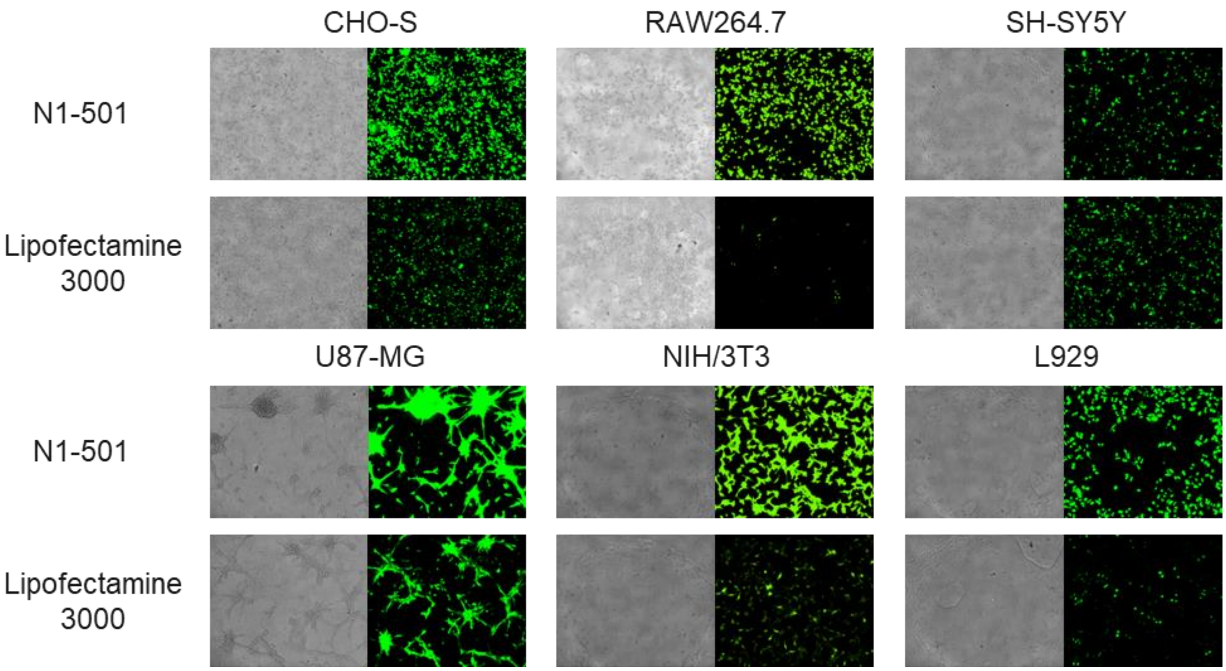

Figure S1. Phase contrast and GFP fluorescent microscopy images of 18 cell lines transfected with N1-501/eGFP mRNA or Lipofectamine 3000/eGFP mRNA nanocomplexes. Pictures were captured 24 hours post-transfection (Scale bar, 400 µm).

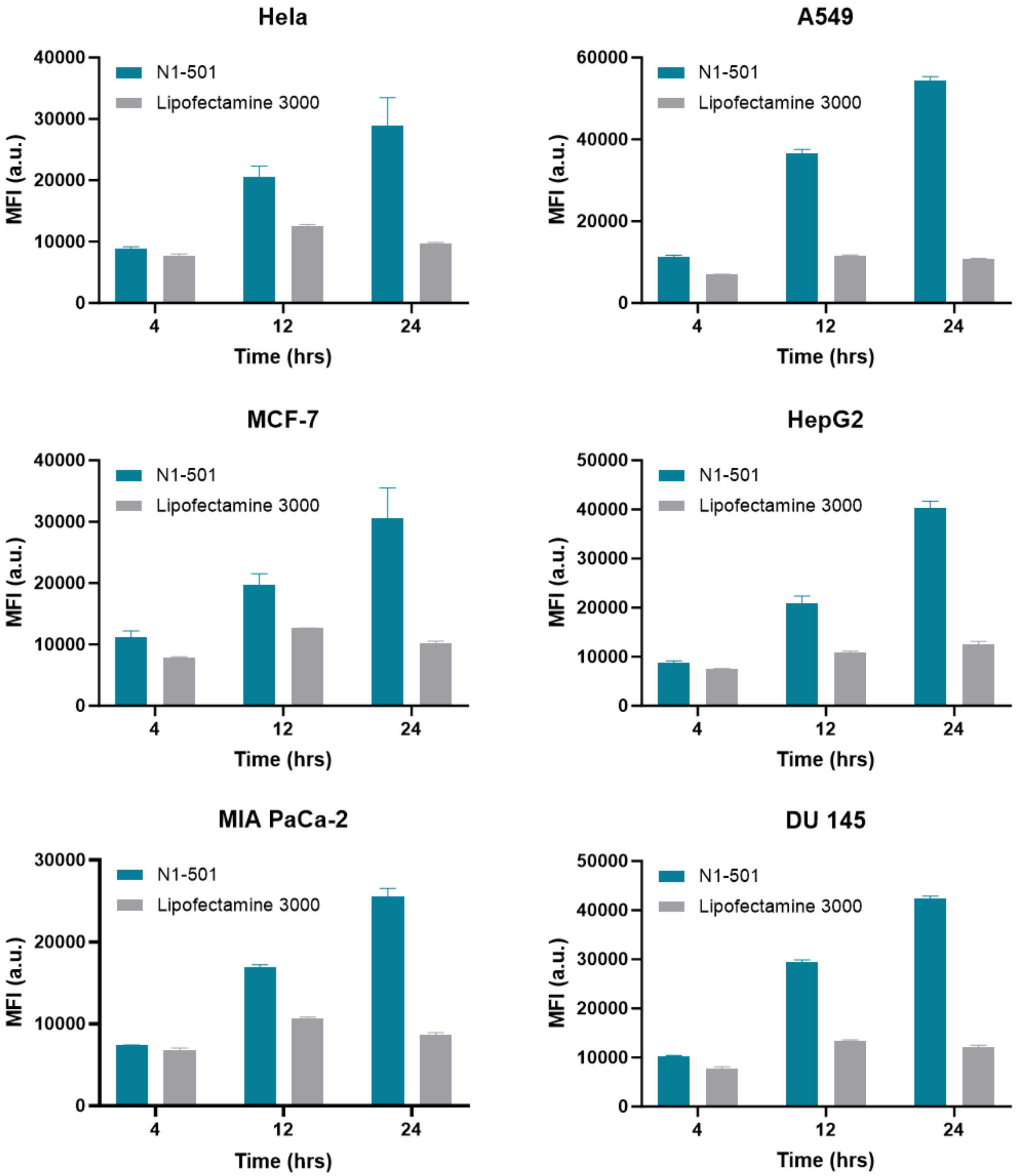
Hela
A549
MCF-7
HepG2
MIA PaCa-2
DU 145
N1-501
Lipofectamine 3000
MFI (a.u.)
Time (hrs)
4
12
24
0
10000
20000
30000
40000
50000
60000

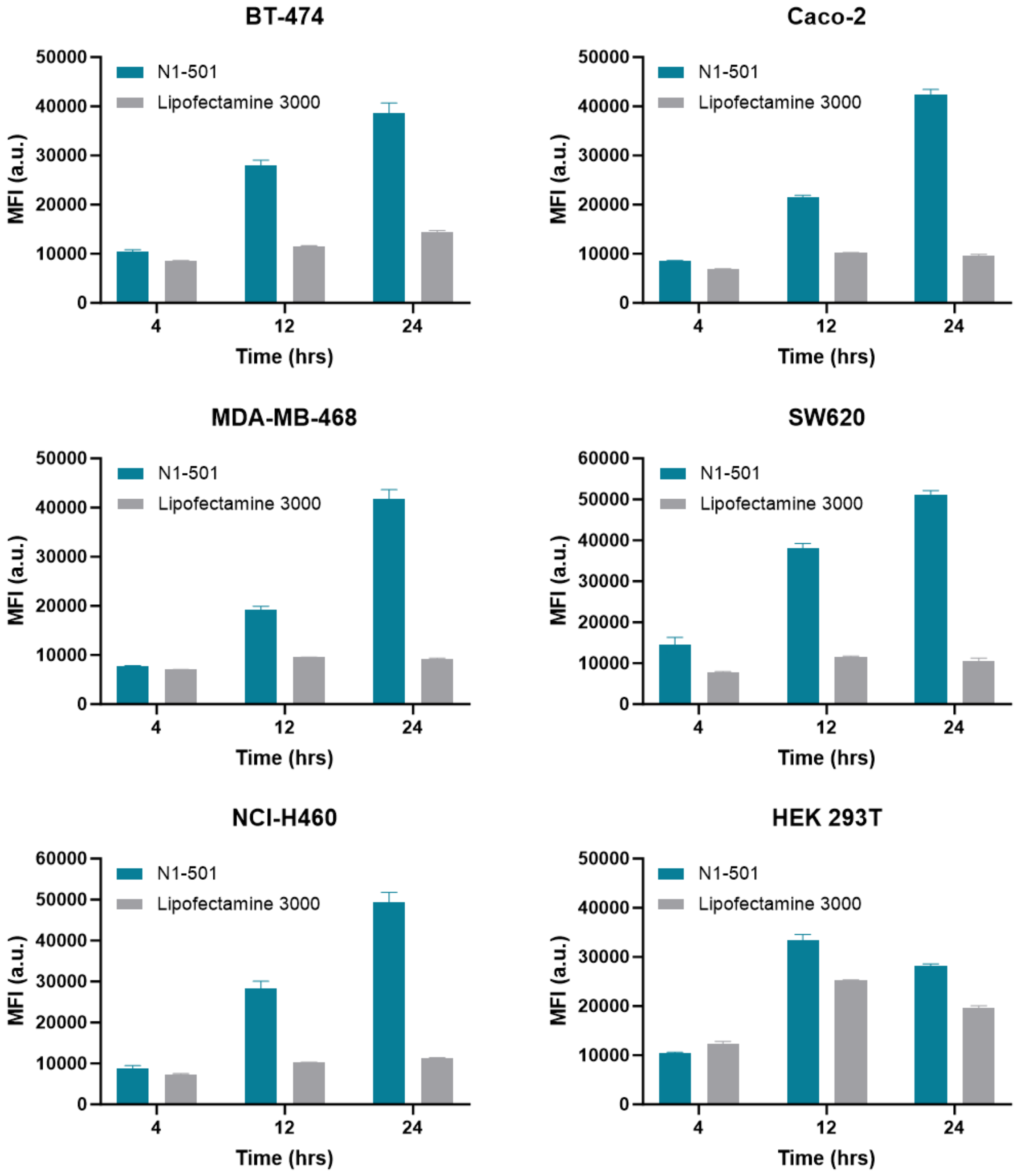
BT-474
Caco-2
MDA-MB-468
SW620
NCI-H460
HEK 293T
N1-501
Lipofectamine 3000
MFI (a.u.)
Time (hrs)
4
12
24
0
10000
20000
30000
40000
50000
60000

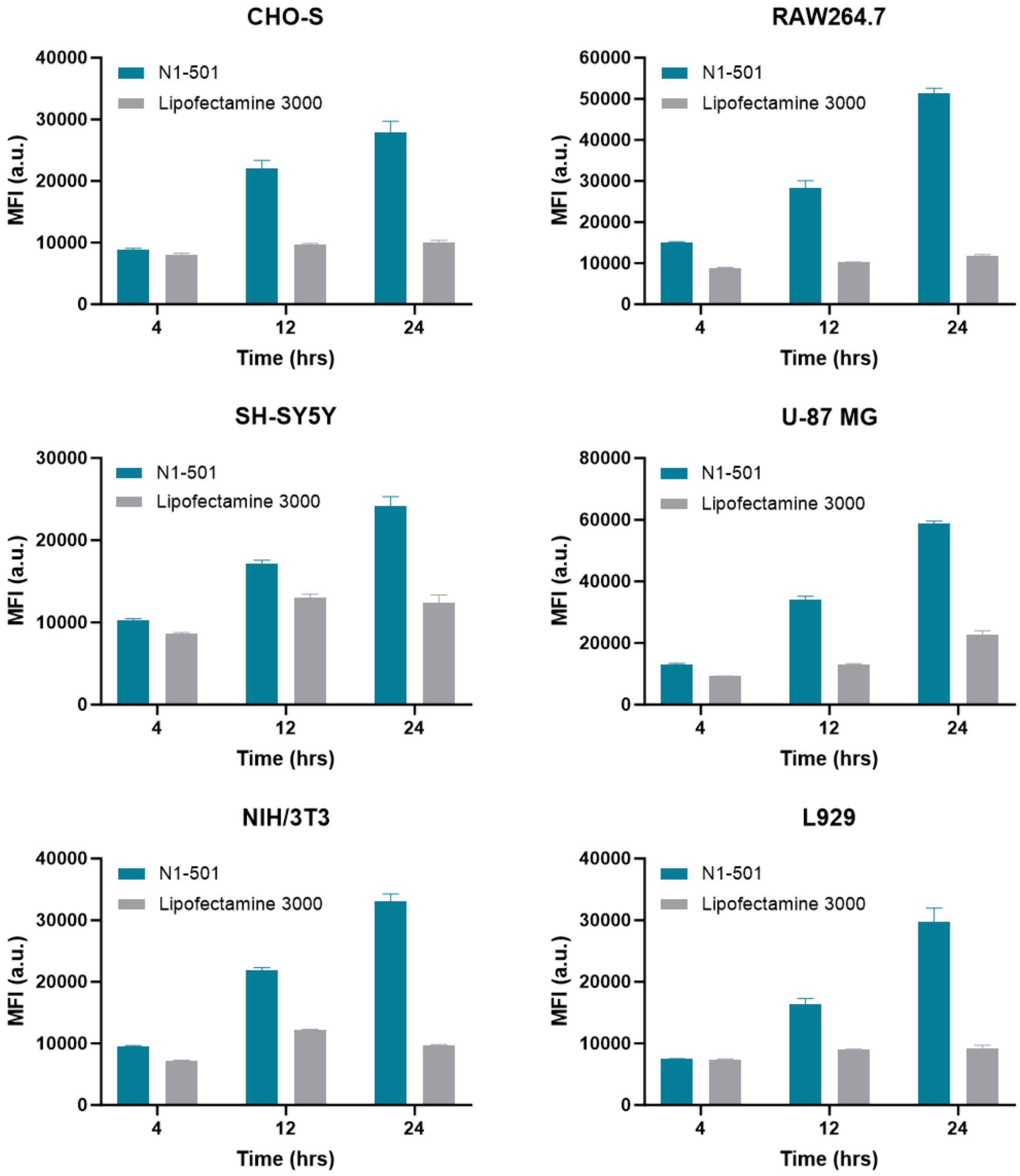

Figure S2. Mean fluorescence intensities (MFI) of 18 cell lines treated with N1-501/eGFP mRNA and Lipofectamine 3000/eGFP mRNA complexes at a mRNA dose of 60 ng and N1-501 dose of 0.30 µL per well. The fluorescence images were captured at 4, 12, and 24 hours post-transfection. The MFI was calculated by dividing the total fluorescence by the number of GFP-positive cells.

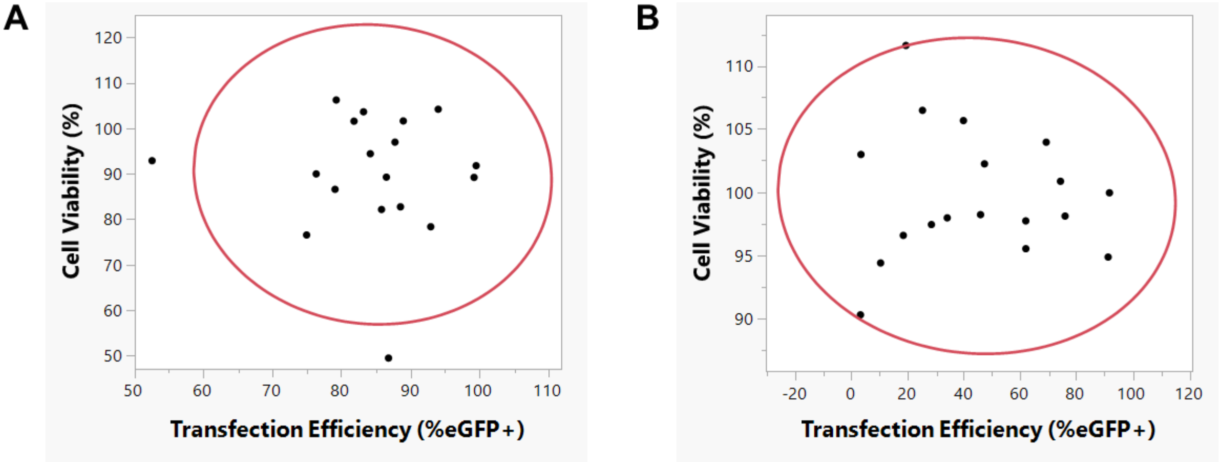

Summary Statistics (A)

	Value	Lower 95%	Upper 95%	Signif. Prob
Correlation	-0.03673	-0.49511	0.437647	0.8850
Covariance	-5.24619			
Count	18			

Summary Statistics (B)

	Value	Lower 95%	Upper 95%	Signif. Prob
Correlation	-0.04554	-0.50175	0.43048	0.8576
Covariance	-6.71042			
Count	18			

Figure S3. The correlation between cell viability and transfection efficiency was analyzed across 18 cell lines transfected with (A) N1-501/eGFP mRNA and (B) Lipofectamine 3000/eGFP mRNA nanoparticles for 24 hours. The summary statistics are shown in the tables below.

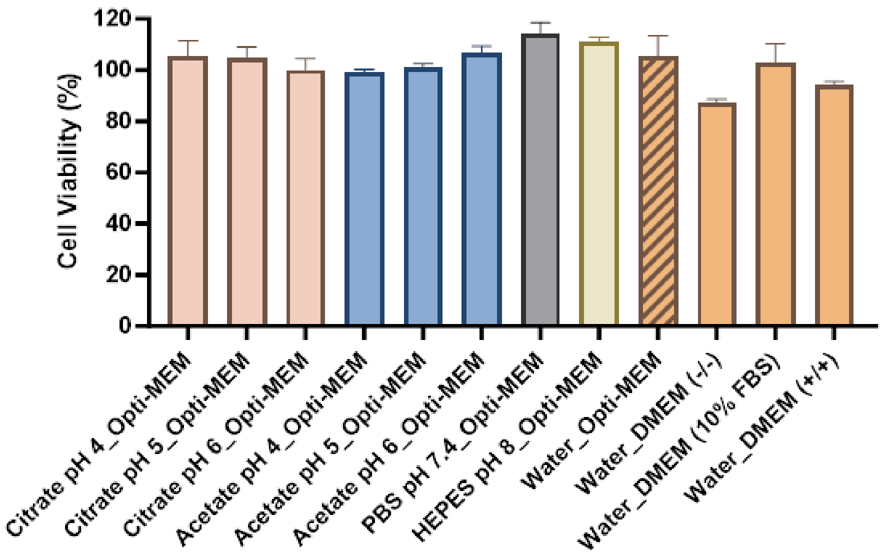

Figure S4. Cell viability of HEK 293T treated with N1-501/eGFP mRNA nanoparticles formulated in different buffers, pH range, and media. Viability was evaluated using CCK-8 assay after 24 hours of treatment.

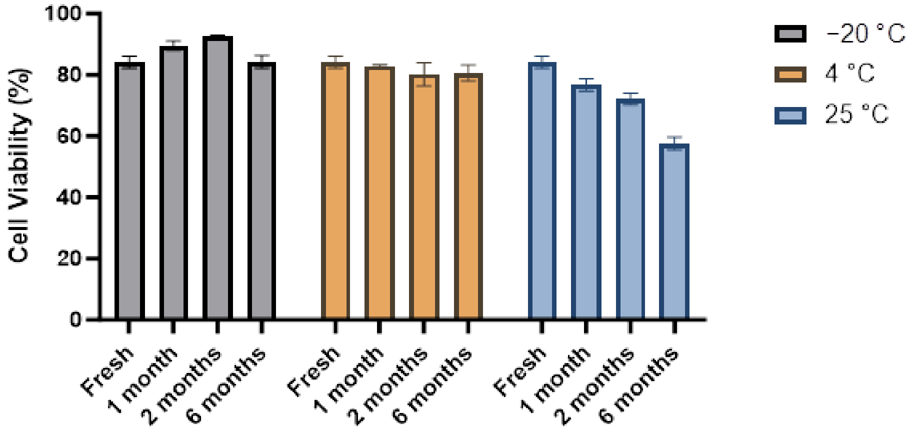

Figure S5. The shelf life of N1-501 was evaluated by transfection efficacy *in vitro*. HEK 293T cell viability was evaluated 24 hours after treatment with N1-501/eGFP mRNA nanoparticles using CCK-8 assay.

Table S1. Cell culture and seeding densities for the 18 cell lines evaluated in this study.

Cell name	Source	Culture medium	Growth properties	Seeding density/well
HeLa	Nanjing RegeneCore Biotech Co.,Ltd	DMEM+10%FBS	adherent	10,000
HEK 293T	Cell Bank/Stem Cell Bank, Chinese Academy of Sciences	DMEM+10%FBS	adherent	15,000
A549	Cell Bank/Stem Cell Bank, Chinese Academy of Sciences	DMEM+10%FBS	adherent	7,500
MCF-7	Vazyme Biotech Co.,Ltd	DMEM+10%FBS	adherent	10,000
HepG2	Nanjing Medical University	DMEM+10%FBS	adherent	7,500
NIH/3T3	Nanjing University	DMEM+10%FBS	adherent	7,500
L929	Zhongmei Guanke Biology Technology Co., Ltd	DMEM+10%FBS	adherent	7,500
MIA PaCa-2	Cell Bank/Stem Cell Bank, Chinese Academy of Sciences	DMEM+10%HI FBS+2.5%HS	adherent	7,500
DU 145	Cell Bank/Stem Cell Bank, Chinese Academy of Sciences	DMEM+10%FBS+1%NEAA	adherent	7,500
BT-474	Jiangsu KeyGEN BioTECH Corp., Ltd	RPMI-1640+10%FBS	adherent	15,000
Caco-2	FuHeng BioLogy	RPMI-1640+10%FBS	adherent	15,000
MDA-MB-468	Cell Bank/Stem Cell Bank, Chinese Academy of Sciences	RPMI-1640+10%FBS	adherent	17,500
SW620	Cell Bank/Stem Cell Bank, Chinese Academy of Sciences	RPMI-1640+10%FBS	adherent	15,000
NCI-H460	Nanjing Cobioer Biosciences CO.,LTD	RPMI-1640+10%FBS	adherent	6,000
RAW264.7	Jiangsu Kanion Pharmaceutical Co., Ltd	DMEM+10%HI FBS	adherent	25,000
SH-SY5Y	Cell Bank/Stem Cell Bank, Chinese Academy of Sciences	DMEM/F12(1:1)+10%FBS	adherent	25,000
U-87 MG	Nanjing RegeneCore Biotech Co.,Ltd	MEM+10%FBS+1%NEAA+1 mM NaP	adherent	15,000
CHO-S	Nanjing RegeneCore Biotech Co.,Ltd	RPMI-1640+10%FBS	non-adherent	15,000

*Abbreviations: Dulbecco's Modified Eagle’s Medium (DMEM), Minimum Essential Media (MEM), Ham's F-12 Nutrient Mixture (F12), RPMI-1640 Medium (RPMI-1640), bovine serum (FBS), heat-inactivated FBS (HI FBS), horse serum (HS), non-essential amino acids (NEAA), sodium pyruvate (NaP).

Table S2. Cell viability of 18 cells treated with N1-501/eGFP mRNA, and Lipofectamine 3000/eGFP mRNA complexes for 24 hours, compared to negative control groups. All studies were conducted using an mRNA dose of 60 ng per well and evaluated with a CCK-8 assay.

Cell name	Cell Viability (%)			
	Untreated cell	eGFP mRNA only	Lipofectamine 3000	N1-501
Hela	100.00±2.18	100.64±4.48	97.73±2.93	103.76±3.82
A549	100.00±14.14	98.25±10.81	99.95±14.68	91.90±11.67
MCF-7	100.00±1.03	103.13±2.07	102.24±1.23	101.70±1.33
HepG2	100.00±5.97	99.38±6.65	98.11±6.99	97.08±6.86
MIA PaCa-2	100.00±8.34	108.92±8.54	111.60±10.43	89.37±6.57
DU 145	100.00±8.03	103.78±9.85	102.98±10.23	101.75±11.17
BT-474	100.00±16.25	90.30±12.83	94.40±14.44	90.09±17.55
Caco-2	100.00±5.09	102.17±5.11	98.22±6.51	76.64±4.24
MDA-MB-468	100.00±6.03	98.37±7.12	97.97±6.57	86.69±4.25
SW620	100.00±4.48	97.83±3.07	95.53±4.37	82.84±4.06
NCI-H460	100.00±6.88	98.44±6.71	97.44±4.48	82.25±5.79
HEK 293T	100.00±3.16	100.26±2.71	94.87±2.38	89.32±2.78
CHO-S	100.00±2.93	109.40±4.20	105.66±4.35	104.32±5.98
RAW264.7	100.00±8.66	100.14±9.69	90.30±7.72	49.54±6.69
SH-SY5Y	100.00±8.23	98.50±20.96	103.95±10.48	93.00±15.18
U-87 MG	100.00±23.63	96.48±19.71	100.86±22.82	106.36±8.65
NIH/3T3	100.00±3.88	101.65±6.17	106.47±5.78	94.53±6.98
L929	100.00±8.14	94.38±6.86	96.58±6.68	78.45±8.07

Table S3. Comparison of the size, polydispersity, and zeta potential of N1-501/eGFP mRNA nanoparticles prepared via pipette mixing and shaker mixing methods. The measurements were conducted using the Malvern Zetasizer Pro.

Sample name	Pipette mixing	Shaker mixing
Size (nm)	125.4±25.0	119.6±13.1
PDI	0.34±0.11	0.20±0.08
Zeta Potential (mV)	29.0±13.2	9.1±3.2
